# Impact of Robotic Technologies on Prostate Cancer Patients’ Choice for Radical Treatment

**DOI:** 10.3390/jpm13050794

**Published:** 2023-05-05

**Authors:** Francesco Esperto, Loris Cacciatore, Francesco Tedesco, Antonio Testa, Pasquale Callè, Alberto Ragusa, Noemi Deanesi, Antonio Minore, Francesco Prata, Aldo Brassetti, Rocco Papalia, Roberto Mario Scarpa

**Affiliations:** 1 Department of Urology, Fondazione Policlinico Universitario Campus Bio-Medico di Roma, 00128 Rome, Italy; francescoesperto@gmail.com (F.E.); francesco.tedesco@unicampus.it (F.T.); antonio.testa@unicampus.it (A.T.); pasquale.calle@unicampus.it (P.C.); alberto.ragusa@unicampus.it (A.R.); noemi.deanesi@unicampus.it (N.D.); antonio.minore@unicampus.it (A.M.); f.prata@unicampus.it (F.P.); rocco.papalia@policlinicocampus.it (R.P.); r.scarpa@policlinicocampus.it (R.M.S.); 2 Department of Urology, IRCCS “Regina Elena” National Cancer Institute, 00128 Rome, Italy; aldo.brassetti@gmail.com

**Keywords:** prostate cancer, ISUP grade, laparoscopic radical prostatectomy, robotic platform, robotic radical prostatectomy

## Abstract

Prostate cancer (PCa) surgery has a strong impact on men’s social and sexual lives. For this reason, many patients ask for robotic surgery. To assess the rate of lost patients due to the lack of a robotic platform (RPl) at our center, we retrospectively selected 577 patients who underwent prostate biopsy between 2020 and 2021 who were eligible for radical prostatectomy (RP) (ISUP ≥ 2; age ≤ 70 yr). Patients eligible for surgery who decided to be operated received a phone call interview asking the reason for their choice. Overall, 230 patients (31.7%) underwent laparoscopic-assisted radical prostatectomy (LaRP) at our center, while 494 patients (68.3%) were not treated in our hospital. Finally, 347 patients were included: 87 patients (25.1%) underwent radiotherapy; 59 patients (17%) were already under another urologist’s care; 113 patients (32.5%) underwent robotic surgery elsewhere; and 88 patients (25.4%) followed the suggestion of friends or relatives based on their surgical experience. Despite no surgical technique for RP having shown superiority in terms of oncological or functional outcomes, patients eligible for PCa treatment decided to be operated on elsewhere because of the lack of an RPl. Our results show how the presence of an RPl may increase the case volume of RP by 49% at our center.

## 1. Introduction

The advent of robotic surgical systems (RSS) in 1999 has had a significant impact on every surgical area, especially urology, gynecology, general surgery, and cardiac surgery. After the first robot-assisted radical prostatectomy (RARP) using the Da Vinci Surgical System^®^ by Binder in 2002 [1], the approach to the radical treatment of localized prostate cancer (LPCa) changed all over the world. This technology combined the minimally invasive advantages of laparoscopic radical prostatectomy (LaRP) with improved surgeon ergonomics and greater technical ease of suture reconstruction of the vesicourethral anastomosis, and has now become the preferred minimally invasive approach when available [2].

In recent years, most patients who come to the hospital for an outpatient visit have a good collection of information regarding their pathologies and possible treatments, which is usually collected through Internet sources and social media platforms such as Instagram, Facebook, YouTube, and TikTok. Specifically, TikTok is one of the fastest growing social media platforms in the urological landscape [3].

PCa surgery has a strong impact on men’s global health, affecting their social and sexual lives. Indeed, after an RP surgical procedure, patients are more worried about potency and continence status as possible surgical complications rather than oncological success. A Cochrane review comparing either RARP or LaRP with open radical prostatectomy (OPR) by including two randomized controlled trials (RCTs) found no significant differences between the comparisons for oncological, urinary, and sexual function outcomes, although RARP and LaRP both resulted in statistically significant improvements in the duration of hospital stay and blood transfusion rates over open RP [4]. Despite this, no surgical approach can be recommended over another [2], while an increasing number of patients ask for novel technologies and robotic surgery. This is probably due to the common belief that what is new is more effective and less invasive.

Regarding oncological results, in 2013 Silberstein et al. published a study comparing early oncologic outcomes of 961 ORP and 493 RARP procedures performed by experienced surgeons in a high volume center; they found that RARP was not associated with lower rates of biochemical recurrence (BCR)-free survival (95.1%, 90.6%, 86.6%, and 81.0% at 1, 3, 5, and 7 years after RARP, respectively) or higher rates of positive surgical margins (PSMs) (8–10% in pT2 disease and 37% in pT3 disease) than other techniques [5,6,7,8,9].

However, two meta-analyses by Ficarra et al. showed a statistically significant advantage in favor of RARP in comparison with both ORP and LaRP in terms of 12-month urinary continence (UI) recovery (4–31%) and in terms of 12-month potency rates (10–46%) [10,11]. In a randomized phase III trial, RARP was shown to have reduced admission times and blood loss but not earlier (12 weeks) functional or oncological outcomes compared to ORP [12], while other subsequent meta-analyses of non-RCTs and RCTs comparing RARP with LaRP have demonstrated that RARP has lower perioperative morbidity and a reduced risk of a positive surgical margin than LaRP, as well as a higher rate of recovery of erectile and continence function [13,14].

The purpose of our study was to assess the rate of patients with a positive prostate biopsy at our institution who decided to be operated on elsewhere because of the lack of a robotic platform (RPl) at our tertiary referral hospital center.

## 2. Materials and Methods

In our study, we evaluated in a retrospective way patients who underwent prostate biopsy at our high-volume tertiary university hospital between January 2020 and December 2021 due to clinical suspicion of PCa, as indicated by a prostate-specific antigen (PSA) value ≥ 4 ng/mL and/or an abnormal digital rectal examination (DRE) and suspicious findings (PI-RADS score ≥ 3) via multiparametric magnetic resonance imaging [2]. Tumor grading was assigned according to the International Society of Urological Pathology (ISUP) criteria [15]. Eligible criteria for RP were an age ≤ 70 years, a clear indication for RP (ISUP ≥ 2), and locally confined disease. Patients with benign prostate hyperplasia (BPH) (*n* = 592) identified with a histopathological report were excluded. Afterwards, we searched for how many patients underwent LaRP at our institution. Surgery was performed by two experienced surgeons (R.P. and G.P.F.).

A phone call interview was performed with all patients eligible for surgery who were not operated on at our center. The phone caller, a urology resident, asked for the reason why the patients decided to be operated on elsewhere; the patients were given the chance to choose between 4 possible options:

1. I wanted to undergo an RARP because I think it can guarantee better results;
2. I was already under another urologist’s care;
3. I preferred radiotherapy because of the fear of surgery and possible complications;
4. Other (friend’s or relative’s suggestion, etc.)

**Figure 1:**
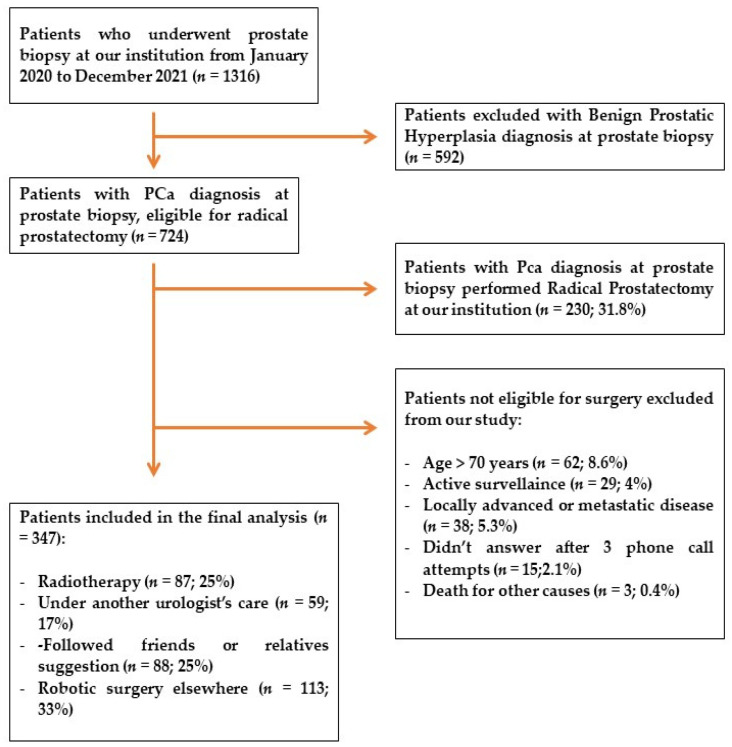
Flowchart of the study.

Figure 1 shows the structure of our study. All answers were collected, and a descriptive statistic was performed with Statistical Package for Social Science software (SPSS version 23, IBM, Chicago, IL, USA).

## 3. Results

**Figure 2:**
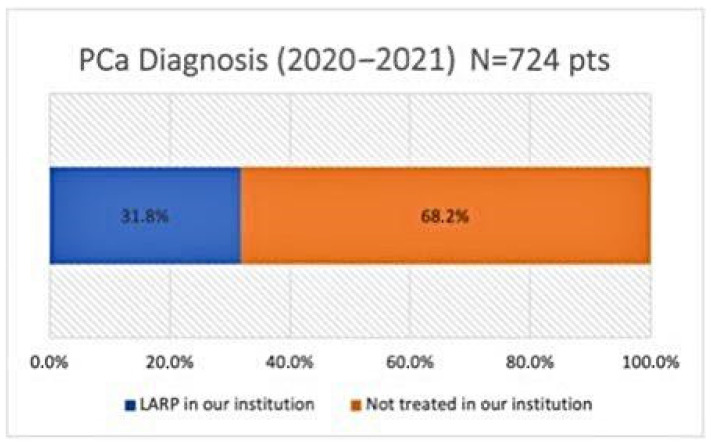
Patients with PCa diagnosis operated on with LARP at our institution versus patients not treated at our institution between January 2020 and December 2021.

During 2020–2021, among the 1316 patients who underwent MRI/US fusion-guided transrectal prostate biopsy at our institution, 724 were diagnosed with PCa with a detection rate of 55%. Concerning patients diagnosed with PCa, 230 patients (31.7%) underwent LaRP at our center (Figure 2). Table 1 shows the exclusion criteria in patients diagnosed with PCa: 62 patients (8.6%) were >70 years; 29 patients (4%) had an ISUP 1 histology and were enrolled in active surveillance protocol; 38 patients (5.3%) were not eligible for RP due to locally advanced or metastatic disease; 15 patients (2.1%) did not answer after 3 phone call attempts; and 3 patients (0.4%) died due to other causes.

**Figure 3:**
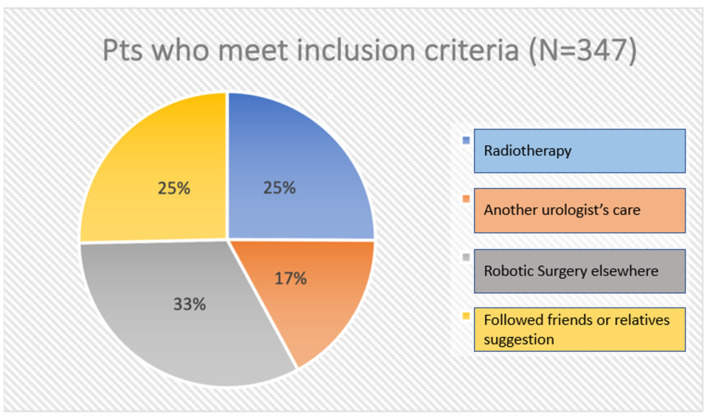
Patients eligible for surgery who were not operated on at our center for different reasons (four possible choices).

Finally, 347 patients were included in our analysis: 87 patients (25%) opted for radiotherapy due to possible consequences of surgery such as urinary incontinence (UI) and erectile dysfunction (ED); 59 patients (17%) declared that they were already under another urologist’s care; 88 patients (25%) declared that they followed the suggestion of friends or relatives to be operated on in other centers because of their previous experience; and 113 patients (33%) felt that robotic technologies may guarantee a better oncological and functional outcome despite their trust in surgical operators and underwent robotic surgery elsewhere. Our results are shown in Figure 3.

## 4. Discussion

With the rise of robotic technology, several studies have investigated the mind changes of patients regarding RP for LPCa and the impact of robotic surgical systems in terms of diffusion, treatment choice, and outcomes compared to the pre-RSS era dominated by oRP and LaRP. Specifically, Schroeck et al. [16], through the administration of a surgical expectation questionnaire to 187 patients undergoing RARP or LaRP, showed how patients who underwent RARP expected a significantly shorter length of hospital stay (OR 0.07, *p* < 0.001), earlier return to physical activity (OR 0.36, *p* = 0.005), and, particularly, a better erectile function (EF) recovery. Another study conducted in 2014 by Egui Rojo et al. [17] analyzed the effect of cosmetic outcome in patients undergoing surgical treatment regarding three variants of RP: oRP (infraumbilical incision and Pfannenstiel incision), LaRP, and RARP; the study consisted of questionnaires accompanied by photographs showing the cosmetic results of these three approaches. Among the 577 patients, the majority (52%) preferred the minimally invasive approach consisting of the six-robotic-port surgery for the treatment of PCa.

Concerning clinical practice, a retrospective multicenter study was carried out by Kobayashi et al. [18] in Japan on 718 patients who underwent RP surgery between 2011 and 2013. Their results revealed an increase rate of 70% for RP after the introduction of a RPl, increasing from 127 cases in 2011 to 221 cases in 2013 (*p* < 0.0001). This evidence relates to both intermediate- or high-risk PCa patients with a mild perioperative risk (ASA score of 2), for which the number of cases increased from 34 to 100, and both low-risk PCa patients with high comorbidity scores (Charlson Index ≥ 4), for which the number of cases increased from 8 to 25; in fact, the introduction of RSS did not affect the distribution of PCa risk as a single factor and did not increase the risk of overtreatment. Moreover, neither expectant management (AS or WW) nor behavior toward PCa diagnosis changed during the clinical adoption of RSS. The impact of RSS on the administration of either radiotherapy (RT) or androgen deprivation therapy (ADT) was statistically significant: indeed, the increase in performing RP brought to a reduction in RT and ADT of 35% in 2011 and 24% in 2013 (*p* = 0.001) and 17% in 2011 and 11% in 2013 (*p* = 0.012), respectively.

Additionally, a community-based study conducted by Neuner et al. [19] reported that RARP had totally replaced retropubic ORP and conventional LaRP three years after its introduction.

The Italian surgical landscape was showed in 2012 by a review article by Santoro and Pansadoro [20]. In 2011, Italy ranked second after the USA, together with Germany, in terms of the number of robotic surgery centers through the rise of the Da Vinci Surgical System. The number of robot-assisted operations in Italy has increased by 25% in one year (4784 in 2010 vs. >6000 in 2011), where the specialty that has benefited the most is urology (2669 robotic interventions in 2010; 56% of all RSS interventions). The urological interventions most affected by RSS concern, first of all, radical prostatectomy, followed by simple nephrectomy, enlarged nephrectomy, partial nephrectomy, pyeloplasty, total and partial cystectomy, adrenalectomies, sacral colpopexy, and pelvic, iliac, and aortocaval lymphadenectomies [21,22]. These Italian results are in line with the findings from the aforementioned studies in Japan due to the spread of robotic technologies. With the robotic platform, the role of the urologic surgeon in the operating room is ever-expanding due to the automation of devices. Nonetheless, prior to operating with robotic surgery techniques, basic robotics skills and procedural tasks must be performed in a safe and effective way as part of robotic training for the surgeons and the residents [23]. Concerning robotic training, Thornblade et al. [24] reported that robotic simulation based on virtual reality will become essential for the adaptation and training of surgeons in the emerging robotic platform landscape. In addition, this robotic training could define the standards to achieve a robotic curriculum.

Despite the rapid increase in robotic surgery in several institutions and the great demand for robot-assisted interventions, the widespread penetration of robotic technology in the public and private healthcare system is still impeded by the high intrinsic costs of the machinery, maintenance, and consumables per procedure; moreover, this technology requires the individual training of medical and nursing staff with the help of a specialized robotic tutor. In this context, the cost is an additional factor to be considered when choosing surgical approaches. Several studies compared the direct costs of different approaches to that of RP. Patient cost is higher for RARP compared to ORP; according to the LAPPRO study in 2018 [25], RARP had the highest direct costs, which may be due to increased surgical instrumentation costs [26]. Only Forsmark et al. [25] reported on the patient cost when comparing RARP and ORP. The mean per patient costs were USD 15,974 (95% CI; 15,405 to 16,543; *p* < 0.001) and USD 12,137 (95% CI; 11,122 to 13,152; *p* < 0.001) in RARP and ORP groups, respectively. The mean difference in terms of cost between RARP and ORP was USD 3837 (95% CI; 2747 to 4928; *p* < 0.001) [22]. In absolute terms, in Italy robotic surgery centers represent a very small minority (46 out of 1000 hospitals, i.e., 4%); furthermore, there are only 116 trained operators out of 35,000 (0.003%) and the number of overall robotic interventions is 6600 out of 35,000,000 (0.002%) procedures [20]. Another recent analysis [27] showed the economic cost of minimally invasive robot-assisted surgery in urology by evaluating RARP and robot-assisted partial nephrectomy (RAPN). In detail, the total cost per patient for RARP was EUR 6857, while it was EUR 6034 per patient for RAPN, which can be divided as 74.1% and 76.2% for surgery costs, 25.9% and 21.5% for hospitalization, and 0% and 2.3% for complications, respectively. Therefore, Grobet-Jeandin et al. demonstrated that RAPN was 6% cheaper and RARP was 10% more expensive per patient compared with an open approach with a reduction in the length of hospital stay.

An updated analysis with follow-up at 24 months did not reveal any significant differences in functional outcomes between the surgical approaches [28]. Increased surgical experience has lowered the complications rate of RP and improved cancer specific survival [29,30,31,32,33,34,35,36,37,38,39]. Lower rates of PMs for high-volume surgeons suggest that experience and careful attention to surgical details can improve cancer control with RP [30,31,32]. There is a lack of studies comparing the different surgical modalities for these longer-term outcomes [33,34,35,36].

A systematic review and meta-analysis of non-RCTs demonstrated that RARP has lower perioperative morbidity and a reduced risk of PMs compared with LaRP, although there was considerable methodological uncertainty [13]. There was no evidence for differences in UI at 12 months and there was insufficient evidence to draw conclusions based on differences in cancer-related, patient-driven, or ED outcomes. Another systematic review and meta-analysis included two small RCTs comparing RARP vs. LaRP [14]. The results suggested higher rates of return of EF (RR: 1.51, 95% CI: 1.19–1.92) and continence function (RR: 1.14, 95% CI: 1.04–1.24) in the RARP group.

Otherwise, a comparative study has also evaluated that there is no clinically relevant difference in cancer control conferred by focal therapy (cryotherapy, high-intensity focused ultrasound, or high-dose-rate brachytherapy) compared to radical therapy (RP with various approaches and radiotherapy) at 6 years [37]. Nonetheless, the development of clear standardized recommendations for RARP survivorship care are essential to improve quality of life and ensure equitable high-quality clinical care [38].

For this reason, after the advent of RSS, numerous studies and systematic reviews focused their attention to try to develop various surgical robotic techniques and post-operative strategies based on RARP to improve UI and ED outcomes, as well as oncological results [40,41,42]. Despite the fact that no surgical approach can be recommended over another, functional and oncological outcomes after RP have been shown to be dependent mostly on surgeons’ experience [28,42] and hospital volume [43]. Nevertheless, the majority of patients require and research the new, that is, the “robotic approach”.

Finally, our results confirm that the lack of an RPl in an institution highlights that patients’ decisions are driven more by the choice of technology than by the choice of surgeon. Additionally, this is due to the wide variety of information readily available from internet sources or social media, which reduces the role of the urologist in the management and surgical choice of the patient with PCa. However, there is a noteworthy rate of the possibility of receiving wrong or untrustworthy information from these sources aforementioned. Despite the high case volume of RP performed at our institution, 33% of patients (*n* = 113) eligible for PCa treatment decided to be operated on elsewhere.

However, our investigation is not devoid of limitations. First of all, our study included a single tertiary referral center. Secondly, we must consider the high availability of RPls in our geographical area as a non-negligible factor that influences patients’ choice. In addition to the DaVinci Robot, with the advent and spread of the Hugo™ RAS System–Medtronic [44,45,46,47] and Versius^®^ Robotic Surgical System [48,49], it would be interesting to effectively assess whether their diffusion and use increase the operating volume for RP and the subsequent costs and earnings for the hospital who bought these novel robotic platforms [50]. Moreover, our study does not report data on the real economic waste regarding each patient lost due to the lack of a robotic system.

Notwithstanding these limitations, to the best of our knowledge, our study is the first to focus its attention on patients’ exodus due to the lack of robotic surgery. Therefore, our study showed how the presence of a robotic surgical platform may increase the case volume of RP by 49% at our center, considering 113 patients were lost who underwent robotic surgery elsewhere versus 230 patients who underwent LaRP at our institution.

## 5. Conclusions

Since the introduction of RSS, there has been a rapid spread of robotic surgery, primarily in the urological area, which has changed the approach to the treatment of LPCa.

Despite RARP being the surgical method most appreciated by operators and patients, it remains a niche surgical method due to the high costs of new technologies, maintenance, and consumables, as well as the high training costs, in a context of national health systems where there is a lack of an equal distribution of novel technologies. For this reason, another study should be carried out with the primary aim of assessing whether the availability of the latest robotic technology with the same surgeons has an impact on patients’ choice.

## Figures and Tables

**Table 1:** Patients not eligible for surgery who were excluded from our study.

Exclusion Criteria after Prostate Biopsy (Patients Diagnosed with PCa)	Patients (*n* = 147)	Patients (%)
Age > 70 years	62	8.6
Active surveillance		
	29	4
	
Locally advanced/metastatic disease		
	38	5.3
Did not answer after 3 phone call attempts		
	
	
	15	2.1
Death due to other causes	3	0.4

## Data Availability

The data reported in this paper are available from the corresponding author upon request. Data is not publicly available due to privacy or ethical restrictions.
